# *GNAS* mutations as prognostic biomarker in patients with relapsed peritoneal pseudomyxoma receiving metronomic capecitabine and bevacizumab: a clinical and translational study

**DOI:** 10.1186/s12967-016-0877-x

**Published:** 2016-05-06

**Authors:** Filippo Pietrantonio, Rosa Berenato, Claudia Maggi, Marta Caporale, Massimo Milione, Federica Perrone, Elena Tamborini, Dario Baratti, Shigeki Kusamura, Luigi Mariani, Monica Niger, Alessia Mennitto, Annunziata Gloghini, Ilaria Bossi, Giulio Settanni, Adele Busico, Pietro Francesco Bagnoli, Maria Di Bartolomeo, Marcello Deraco, Filippo de Braud

**Affiliations:** Medical Oncology Department, Fondazione IRCCS Istituto Nazionale dei Tumori, Via Venezian, 1, 20133 Milan, Italy; Pathology and Molecular Biology Department, Fondazione IRCCS Istituto Nazionale dei Tumori, Milan, Italy; Surgery Department, Fondazione IRCCS Istituto Nazionale dei Tumori, Milan, Italy; Unit of Medical Statistics, Biometry and Bioinformatics, Fondazione IRCCS Istituto Nazionale Tumori, Milan, Italy; Department of Cancer Surgery, Humanitas Clinical and Research Center, Milan, Rozzano Italy

**Keywords:** Peritoneal pseudomyxoma, Appendiceal cancer, Metronomic capecitabine, Bevacizumab, Next-generation sequencing, *GNAS*

## Abstract

**Background:**

There is lack of evidence about systemic treatment of pseudomyxoma peritonei (PMP) relapsing after cytoreductive surgery and hyperthermic intraperitoneal chemotherapy. There is also lack of biomarkers able to predict outcomes beyond known clinical and pathological prognostic features.

**Methods:**

Fifteen patients with relapsed PMP and progressive disease within the last 6 months were included and received metronomic capecitabine (625 mg/mq/day b.i.d.) and bevacizumab (7.5 mg/Kg three-weekly) until progressive disease/unacceptable toxicity. The primary endpoint was progression-free survival (PFS). Ion Torrent^®^ next generation sequencing technology (Hot-spot Cancer Panel) was used to characterize molecular features.

**Results:**

At a median follow up of 12 months, median PFS was 8.2 months and 1-year overall survival was 91 %. Partial responses were observed in 20 % of cases, but a significant reduction of tumor markers in up to 79 %. Treatment was very well tolerated without no new safety signals. All tumor samples except one had *KRAS* mutations. Patients with *GNAS* mutations had a significantly shorter median PFS as compared to *GNAS* wild-type ones (5.3 months vs. not reached; p < 0.007). The results were externally validated on our previous series of PMP patients. *GNAS* mutations were rare in a parallel cohort of 121 advanced colorectal cancers (2.5 %), but were associated with peculiar clinical-pathological features and aggressive course.

**Conclusions:**

Metronomic capecitabine and bevacizumab is an active and well tolerated option in patients with relapsed PMP. The negative prognostic effect of *GNAS* mutations in gastrointestinal cancers warrants further confirmatory studies and may prompt the development of effective targeted strategies.

**Electronic supplementary material:**

The online version of this article (doi:10.1186/s12967-016-0877-x) contains supplementary material, which is available to authorized users.

## Background

Pseudomyxoma peritonei (PMP) has an incidence of 1–2 per million/year, and consists in accumulation of mucinous peritoneal implants usually arising from appendiceal neoplasms [1]. Independently from its low or high histological grade [2, 3], PMP is initially managed with cytoreductive surgery (CRS) and hyperthermic intraperitoneal chemotherapy (HIPEC), with the goal of extirpating both gross and microscopic disease, respectively. This approach confers a 20-years overall survival (OS) greater than 70 % [4, 5], comparing favourably with historical controls. However, a significant proportion of patients will ultimately die of loco-regional disease progression [5]. While repeated CRS may confer some benefit to selected patients, there is a lack of evidence about systemic treatment of unresectable or relapsed PMP. Despite a borderline malignant potential and low proliferation index, non-randomized series reported encouraging results with fluoropyrimidine-based combination chemotherapy [6, 7].

The anti-vascular endothelial growth factor (VEGF) monoclonal antibody bevacizumab is routinely used in advanced colorectal cancer, as well as in peritoneal carcinomatosis secondary to ovarian cancer [8, 9]. Metronomic therapy with low-dose continuous administration of chemotherapy in combination with bevacizumab may be effective in colorectal and ovarian cancers [10, 11]. Bevacizumab and metronomic capecitabine may have synergic antiangiogenic, immunomodulatory and cytostatic effects, being a useful strategy for slow-growing and relatively resistant tumors such as PMP.

In this mono-institutional study, we aimed at assessing the efficacy and safety of metronomic capecitabine and bevacizumab as palliative treatment of patients with relapsed PMP.

## Patients and methods

### Patients

Patients with low or high-grade PMP of appendiceal origin were eligible. Inclusion criteria were: age ≥18 years; disease relapse following CRS and HIPEC, AND progressive disease (PD) assessed by computed tomography scan during the last 6 months; ECOG performance status 0–1; adequate bone marrow, hepatic, and renal functions. Exclusion criteria were: prior systemic chemotherapy; plan of repeated surgery in case of response to treatment or after a neoadjuvant treatment phase; uncontrolled hypertension; clinically significant cardiovascular disease; coagulopathy; history of malignancy in the previous 3 years.

The study was conducted according to Good Clinical Practices and was approved by the local ethics committee (study INT 14/14). All subjects provided written informed consent prior to study procedures.

### Study flowchart

Patients received oral capecitabine 625 mg/sqm/day (≥70 years: 500 mg/mq/day) b.i.d. plus intravenous three-weekly bevacizumab 7.5 mg/kg, until progressive disease or unacceptable toxicity.

The primary study endpoint was progression-free survival (PFS). Secondary endpoints were: OS; overall response rate (ORR) according to RECIST vers. 1.1 criteria. [12]; tumor markers response; safety.

Baseline evaluations included the following: semiology; full blood tests; electrocardiogram; computed tomography (CT) scan of the chest and abdomen; all circulating tumor markers CEA, CA 19-9, CA 125 and CA15-3. During treatment, adverse events (AEs) were assessed according to NCI-CTC vers. 4.03 [13]. CT scans and tumor markers were repeated every 12 weeks until PD.

### Biomarkers evaluation

Formalin-fixed paraffin-embedded tissues of peritoneal deposits were reviewed for tumor content and manually microdissected to isolate a high percentage of neoplastic cells. DNA was isolated using the GeneRead DNA FFPE kit (Qiagen, Hilden, Germany, http://www.qiagen.com Cat. n. 180134). Next generation sequencing of 50 genes’ hotspot regions included in the Hotspot Cancer Panel v2 (Life Technologies) was then performed by using the Ion Torrent Personal Genome Machine platform (Life Technologies) [14]. Additionally, *MGMT* promoter methylation status was assessed by methylation specific polymerase reaction, as previously described [15]. *MET* amplification status was assessed by bright field dual-color SISH analysis (Ventana Medical Systems) in at least 40 non overlapping nuclei; MET expression by using a rabbit monoclonal anti-MET antibody (dilution 1:200; clone SP44, Spring Bioscience, Pleasanton, CA) (Additional file 1: Methods S1) [16].

### Statistical analysis

The study was designed to show non–inferiority toward historical experience, in which median progression free survival is around 8 months. Accordingly, non-inferiority of the experimental treatment was inferred if the lower boundary of the 95 % confidence interval (CI) exceeded 5 months, a threshold clearly denoting a detrimental effect. With this rule, we estimated that a sample size of 15 patients yields 80 % power in case of a median PFS of 10 months, a value chosen to reflect a hypothetical improvement with the experimental treatment.

All time-to-event parameters were calculated by the Kaplan–Meier method. Median values were calculated and presented with 95 % confidence intervals. Data were analysed using the SAS System for Windows, version 9.2.

## Results

### Patients population

From February 2014 to July 2015, fifteen patients were included. The data lock was in October 2015. Baseline demographic and disease characteristics are shown in Table 1. Briefly, one-third of the patients had high grade histology and 47 % had relapse within 1 year from CRS/HIPEC. All patients except one had baseline elevation of at least one tumor marker.

**Table 1:** Patients and disease characteristics

Main characteristics (N = 15)	N° (%)
Age, years	
Median (range)	52 (42–68)
Sex	
Male	9 (60)
Female	6 (40)
ECOG performance status	
0	12 (80)
1	3 (20)
Histological grade	
High	5 (33)
Low	10 (67)
Time from CRS + HIPEC to relapse	
Median (range, months)	13 (5–36)
≤12 months	7 (47)
>12 months	8 (53)
PCI at the time of CRS + HIPEC	
Median (range)	27 (8–39)
Completeness of cytoreduction	
CC-0	8 (53)
CC-1	7 (47)

*CRS* cytoreductive surgery; *HIPEC* hyperthermic intraperitoneal chemotherapy; *PCI* peritoneal carcinomatosis index; *CC* completeness of cytoreduction score

### Efficacy outcomes

**Fig. 1:**
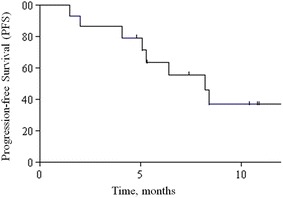
Kaplan–Meier curve for progression-free survival

**Fig. 2:**
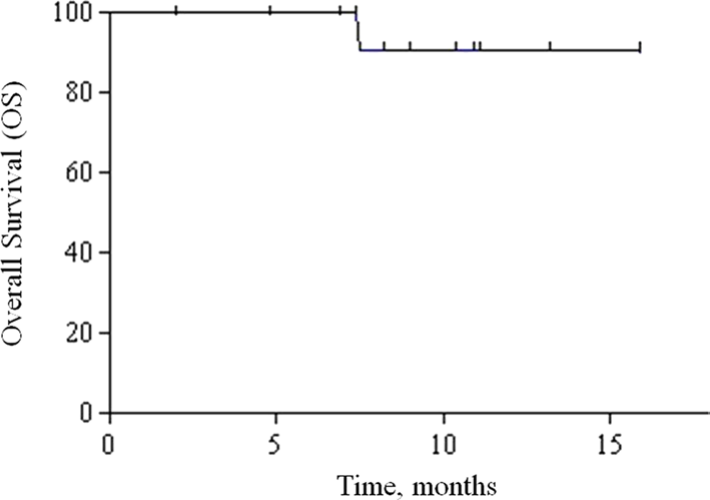
Kaplan–Meier curve for overall survival

At a median follow-up of 12 months (range 3–18), 8 (53 %) patients had PD and only 2 died. The primary endpoint was not inferior to historical controls, with a median PFS of 8.2 months (95 % CI 5.3-not assessable). Obviously, median OS was not reached, since the 1-year OS rate was 91 % (95 % CI, 75 %-100 %). Kaplan–Meier curves for PFS and OS of the study population are depicted in Figs. 1 and 2, respectively. Concerning secondary endpoints of activity, three patients had a PR, accounting for an ORR of 20 %. At the time of this analysis, two responses were still ongoing (12+ months and 18+ months). Ten (67 %) patients had a SD and only 2 (13 %) had early PD. Clinical benefit rate (CR + PR + SD) was 87 %. Reductions of in the tumour marker levels by more than 30 % were recorded for 11 (79 %) of 14 evaluable patients.

### Safety outcomes

All patients received at least one treatment cycle and were evaluable for safety. Median number of cycles was 6 (range 3–18). The percentages of patients who experienced at least one AE were: 53 % (grade 3 or 4: 13 %). Table 2 depicts the frequency of all grades of AEs during treatment. Treatment discontinuation related to serious AEs was reported in 1 case, while dose reductions of capecitabine were performed in only 2 cases. The only treatment-related grade 3/4 AEs were hypertension, thromboembolism and hand-foot syndrome—while other AEs were mild to moderate (mainly diarrhoea and fatigue).

**Table 2:** Treatment-related toxicity

Adverse events	N° of patients (%) grade according NCI-CTC vers. 4.03			
	G1	G2	G3	G4
Hypertension	–	1 (6 %)	1 (6 %)	–
Thromboembolic event	–	1 (6 %)	1 (6 %)	–
Diarrhea	2 (13 %)	1 (6 %)	–	–
Neutropenia	–	1 (6 %)	–	–
Hand Foot Syndrome	–	1 (6 %)	1 (6 %)	–
Epistaxis	1 (6 %)	–	–	–
Fatigue	2 (13 %)	–	–	–

*NCI*-*CTC, National Cancer Institute*—*Common Toxicity Criteria*

### Biomarkers

As shown in Table 3, all 15 peritonectomy samples were available. In the next-generation sequencing analysis, sequence reads were filtered for quality: in the variant caller plugin, we included the variant that had a quality score at least 20. Eighty-five percent of the bases called by PGM have quality phred-scores of Q30 (A phred score of Q30 corresponds to a probability of base-call error of 1 in 1000 or 99.9 % accuracy). For all sample, the average base coverage depth was at least 1500× and uniformity at least 94 %.

**Table 3:** Genomic alterations detected in the 15 samples

ID	Mutations detected by NGS												
	Tumor content (%)	*KRAS* mutation	Mutant alleles (%)	Mutant alleles normalized for tumor content (%)	HS	GNAS mutation	Mutant alleles (%)	Mutant alleles normalized for tumor content (%)	HS	Other mutations (mutant alleles; normalized for tumor content; HS)	MGMT methylation^a^	MET Expression^b^	MET amplification^c^
1	45	G12C	12	27	54	R201H	16	36	72		No	300	No
2	40	G12D	26	65	130	R201H	24	60	120		Yes	100	No
3	30	WT	–	–	–	WT	–	–	–		No	60	No
4	40	G12D	12	30	60	R201H	11	28	56		No	80	2 % small clusters
5	20	G13D	7	35	70	WT	–	–	–	TP53 R248 W (20 %;100 %; 200)	No	250	>5 signals in < 50 % cells
6	10	G13D	4	40	80	WT	–	–	–		Yes	180	No
7	20	G12D	9	45	90	WT	–	–	–	FGFR3 A257 V (9 %; 45 %; 90) LKB1 P319S (8 %; 40 %; 80)	No	90	No
8	50	G12D	13	26	52	R201C	14	28	56	TP53 R273H(23 %; 46 %; 92) HNF1A R278 W (5 %; 10 %; 20)	No	10	No
9	50	G12D	30	60	120	R201H	15	30	60		Yes	140	No
10	30	G12 V	8	27	54	R201H	3	10	20		No	160	1 % small clusters
11	20	G12 V	18	90	180	WT	–	–	–	TP53 C238F (19 %; 95 %; 190)	No	60	NO
12	10	G12D	1	10	20	R201H	6	60	120		No	90	No
13	35	G12V	5	14	28	R201H	10	29	58		No	90	No
14	80	G12D	23	29	58	R201H	24	30	60		No	60	No
15	30	G12D	4	13	26	WT	–	–	–		No	140	No

^a^ MGMT methylation status is assessed by methylation-specific Polymerase chain reaction

^b^ MET expression is assessed by immunohistochemistry and is reported as H-score

^c^ MET amplification status is assessed by in situ hybridization

**Fig. 3:**
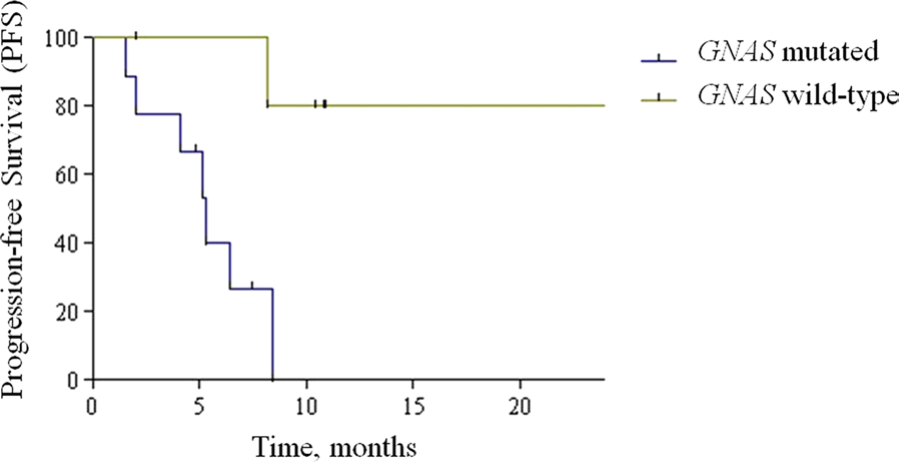
Comparison of Kaplan-Meier curves for progression-free survival according to *GNAS* mutational status in the prospective cohort (metronomic capecitabine and bevacizumab)

Given the low cellularity of these tumors, the Normanno et al. [17] heterogeneity score of each mutation was too wide to allow comparisons with colorectal cancer NGS data. All tumors except one (93 %) had *KRAS* exon 2 mutations (80 % in codon 12; 13 % in codon 13), while no *NRAS* or *BRAF* mutations were detected. *GNAS* mutations were found in 9 (60 %). No significant associations between *GNAS* mutations and the clinic-pathological variables (including age, gender, ECOG performance status, histological grade, time elapsed from surgery to relapse, PCI and completeness of cytoreduction) were found. However, median PFS was significantly shorter in patients with *GNAS* mutations as compared to those with *GNAS* wild-type status (5.3 months versus not reached; Hazard Ratio [HR] for progression 7.57, 95 % CI, 1.73–33.20; log-rank test p = 0.007; Fig. 3). The two tumors with early PD had a *GNAS* mutation. In attempt to externally validate *GNAS* mutations as prognostic, we explored their interaction with PFS in FOLFOX-4 treated patients included in our previous paper [6]. Of 11 available samples, *GNAS* mutations were found in 6 (55 %), were not associated with clinic-pathological variables, but maintained a similar prognostic effect (log-rank test p = 0.002; Additional file 2: Figure S1). Coupling together the two series, we obtained a pooled dataset of 26 patients, of whom 15 (58 %) had a *GNAS* mutation. We were able to confirm that median PFS was significantly shorter in patients with *GNAS* mutation as compared to those with *GNAS* wild-type status (5.1 versus 13 months; HR for progression 11.26, 95 % CI 3.62–35.06; log-rank test p < 0.0001; Additional file 3: Figure S2).

Regarding ancillary data, *TP53* mutations were found in 3 (20 %) samples, while *HNF1A* was mutated in 1 case, and *FGFR3* plus *LKB1* were found concomitantly mutated in a patient with long-lasting benefit. *MGMT* promoter methylation was found in 3 (20 %) samples. *MET* gene was never amplified and MET protein was overexpressed in 2 (13 %) cases.

Finally, we sought to investigate the role of *GNAS* mutations in patients with metastatic colorectal cancer. By using the same next generation sequencing technology (Ion Torrent®) in a retrospective mono-institutional series of 121 cases, we found *GNAS* mutation in only 3 (2.5 %). Surprisingly, all cases shared the same peculiar clinical and pathological features: right side origin, mucinous histology, synchronous peritoneal metastases and concomitant *KRAS* mutations. Additionally, all three patients had a PD as best response to both FOLFOX and FOLFIRI regimens, even when combined with bevacizumab (Additional file 4: Table S1).

## Discussion

In patients with unresectable or recurrent PMP of appendiceal origin, fluoropyrimidine-based combination chemotherapy may be helpful for palliation of symptoms and slowing disease progression [17]. In a prospective cohort of 20 patients treated with FOLFOX-4 regimen, we obtained an ORR of 20 % and a median PFS of 8 months [6]. A previous phase II study evaluated the combination of capecitabine with mitomycin-C, even if only 22 evaluable patients had progressive disease at baseline [7]. In this subgroup, ORR was 14 %, while PFS was not reported. This scenario raises two fundamental issues. First, the improvement of medical treatment of this orphan disease is an urgent and unmet clinical need. Second, the availability of robust evidences is limited by the rarity of the disease, the heterogeneity of clinical, pathological and biological features, and variability of natural history—ranging from an extremely indolent course (for which watchful waiting is still a valid option) to a frankly malignant one. In our study, all patients had progressive disease within 6 months prior to study entry, and not surprisingly showed a significant proportion of adverse prognostic features, such as high grade histology or time to relapse from CRS and HIPEC <12 months. The combination of metronomic capecitabine and bevacizumab achieved results comparable to prior experiences, since ORR was 20 % and median PFS was 8.2 months. Importantly, the safety profile was extremely favourable, making the combination as a valuable treatment option with a good therapeutic index. The reasons for investigating this combination were several. First, the possibility to continue treatment until PD with minimal chronic toxicity is a fundamental difference with oxaliplatin- or mitomycin-C-based combinations [18]. Second, metronomic therapy may be active through multiple mechanisms, such as restoring of anticancer immune response, induction of tumor dormancy, and targeting tumor angiogenesis [19–23]. Finally, especially considering slow-proliferating tumors such as PMP, the metronomic schedule of capecitabine administration may achieve a long-lasting cytostatic effect and synergize with anti-VEGF agents, such as bevacizumab [24].

We shared new insights into the molecular landscape of PMP and appendiceal cancers [25]. First, the identification of new treatment targets in this orphan disease is strictly dependent on a better knowledge of its biological features. Second, clinicians should be aware the natural history of PMP is not only influenced by validated prognostic factors [5, 26, 27]—such as completeness of cytoreduction, peritoneal carcinomatosis index and histological grade—but also by potential prognostic biomarkers linked to disease aggressiveness. The prevalence of *GNAS* mutations in our study was in line with the literature data [28, 29]. Exploiting our homogeneous data-set, we were able to show for the first time a potential prognostic effect of such mutations following disease relapse and palliative treatment with fluoropyrimidine-based chemotherapies. Interestingly, we confirmed a low prevalence (2.5 %) of *GNAS* mutations in colorectal cancer, similarly to what was previously described [30]. However, our retrospective large cohort of patients seemed to identify *GNAS*-mutated colorectal cancer as associated with aggressive clinical course and phenotypic characteristics resembling to PMP (right side origin, mucinous histology, peritoneal involvement and concomitant *KRAS* mutations). However, evidence on the pathogenetic and prognostic role of GNAS mutations in colorectal cancer cannot be considered definitive given their rarity and the retrospective nature of our data.

We confirmed that the prevalence of *KRAS* mutations in PMP was among the highest in human pathology, as highlighted by previous studies using highly sensitive techniques [28]. However, in the largest available series of PMP, *KRAS* mutations were not prognostic following curative surgery [31]. It is important to point out that the classical histology of PMP is constituted by sparse neoplastic cells within a background of abundant mucinous deposits. Thus, standard direct DNA sequencing techniques may be not sensitive enough to detect low amounts of cells harbouring mutations and may represent the cause of frequent false negative results. Mutant-enriched polymerase chain reaction or next-generation sequencing methods may allow the detection of low levels of mutant DNA against a background of wild-type sequence and is of particular value for the molecular characterization of tumors with low cellularity such as PMP [28, 31, 32]. Not surprisingly, the Normanno et al. [17] heterogeneity score was quite wide in our samples.

Our study has some obvious limitations. Both the small sample size and lack of randomization make impossible to quantify the relative benefits from the addition of bevacizumab to fluoropyrimidines. For the same reasons, it is impossible to distinguish between the poor prognostic effect of *GNAS* mutations and their potential value as predictive biomarker of treatment resistance.

In conclusion, metronomic capecitabine and bevacizumab is an active and well tolerated combination in patients with relapsed PMP. The negative prognostic effect of *GNAS* mutations warrants further confirmatory studies and may prompt the development of effective targeted strategies.

## Conclusions

In this study, 15 patients with relapsed and progressive peritoneal pseudomyxoma were treated with metronomic capecitabine and bevacizumab, achieving a 20 % response rate and a 8.2 months progression-free survival. A next-generation sequencing approach identified *GNAS* mutations as prognostic biomarker in this homogeneous population. Patients with *GNAS* mutations had significantly poorer outcome in terms of progression-free survival.

This observation was externally validated in a previous patients series of peritoneal pseudomyxoma patients treated with FOLFOX-4 chemotherapy. Additionally, the prognostic role of *GNAS* was also explored in a retrospective data-set of 121 advanced colorectal cancers: *GNAS* mutations seemed to identify a new and rare molecular subtype with unfavorable outcome and peculiar clinical and pathological characteristics—such as right side origin, mucinous histology, peritoneal involvement and concomitant *KRAS* mutation.

## Additional files

10.1186/s12967-016-0877-x MET assessment.
10.1186/s12967-016-0877-x Comparison of Kaplan-Meier curves for progression-free survival according to *GNAS* mutational status in the retrospective cohort (FOLFOX-4).
10.1186/s12967-016-0877-x Comparison of Kaplan-Meier curves for progression-free survival according to *GNAS* mutational status in both the prospective (metronomic capecitabine and bevacizumab) and the retrospective cohort (FOLFOX-4).
10.1186/s12967-016-0877-x Genomic alterations detected in the 3 GNAS mutated colorectal cancer and matched clinical-pathological data.
