# Supplementary material for: GNAS mutations as prognostic biomarker in patients with relapsed peritoneal pseudomyxoma receiving metronomic capecitabine and bevacizumab: a clinical and translational study
Source: J Transl Med. 2016 May 6;14:125. doi: 10.1186/s12967-016-0877-x (PMC4859944; doi:10.1186/s12967-016-0877-x)
Supplement: Supplementary file 1 — 10.1186/s12967-016-0877-x MET assessment. [file 12967_2016_877_MOESM1_ESM.docx]

***Supplementary Material, Methods***

*MET* gene amplification was defined as positive when: a) *MET*/CEP7 ratio was > 2 or b) average number of *MET* signals per tumor cell nucleus was > 6, or c) tumor cells containing > 5 signals were > 50% of tumor cells, or d) tumor cells containing > 5 signals with a ratio > 2 were > 10% of tumor cells. Small or large clusters were considered to be 6 signals and 12 signals, respectively. MET protein immunohistochemical expression was evaluated according to a semi-quantitative assessment (H-score) which multiplies staining intensity (scored from 0 to 4) with percentage of positive cells (scored 0–100%). Scores from 0 to 200 are considered negative/low expression and scores from 201 to 400 are considered positive/high expression.
