# Supplementary figures and images for: GNAS mutations as prognostic biomarker in patients with relapsed peritoneal pseudomyxoma receiving metronomic capecitabine and bevacizumab: a clinical and translational study
Source: J Transl Med. 2016 May 6;14:125. doi: 10.1186/s12967-016-0877-x (PMC4859944; doi:10.1186/s12967-016-0877-x)

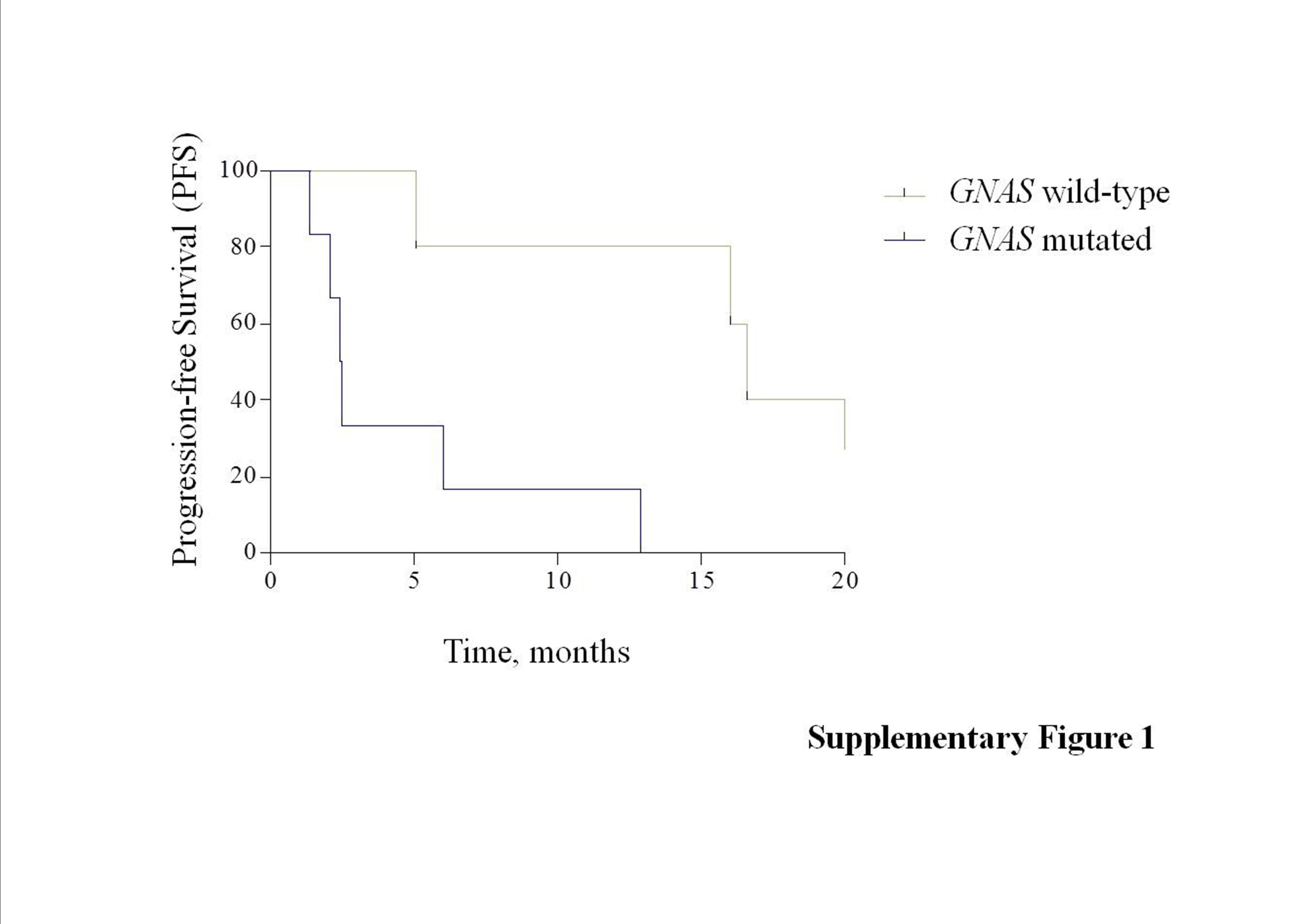

Supplement: Supplementary file 2 — 10.1186/s12967-016-0877-x Comparison of Kaplan-Meier curves for progression-free survival according to GNAS mutational status in the retrospective cohort (FOLFOX-4). [file 12967_2016_877_MOESM2_ESM.jpg]

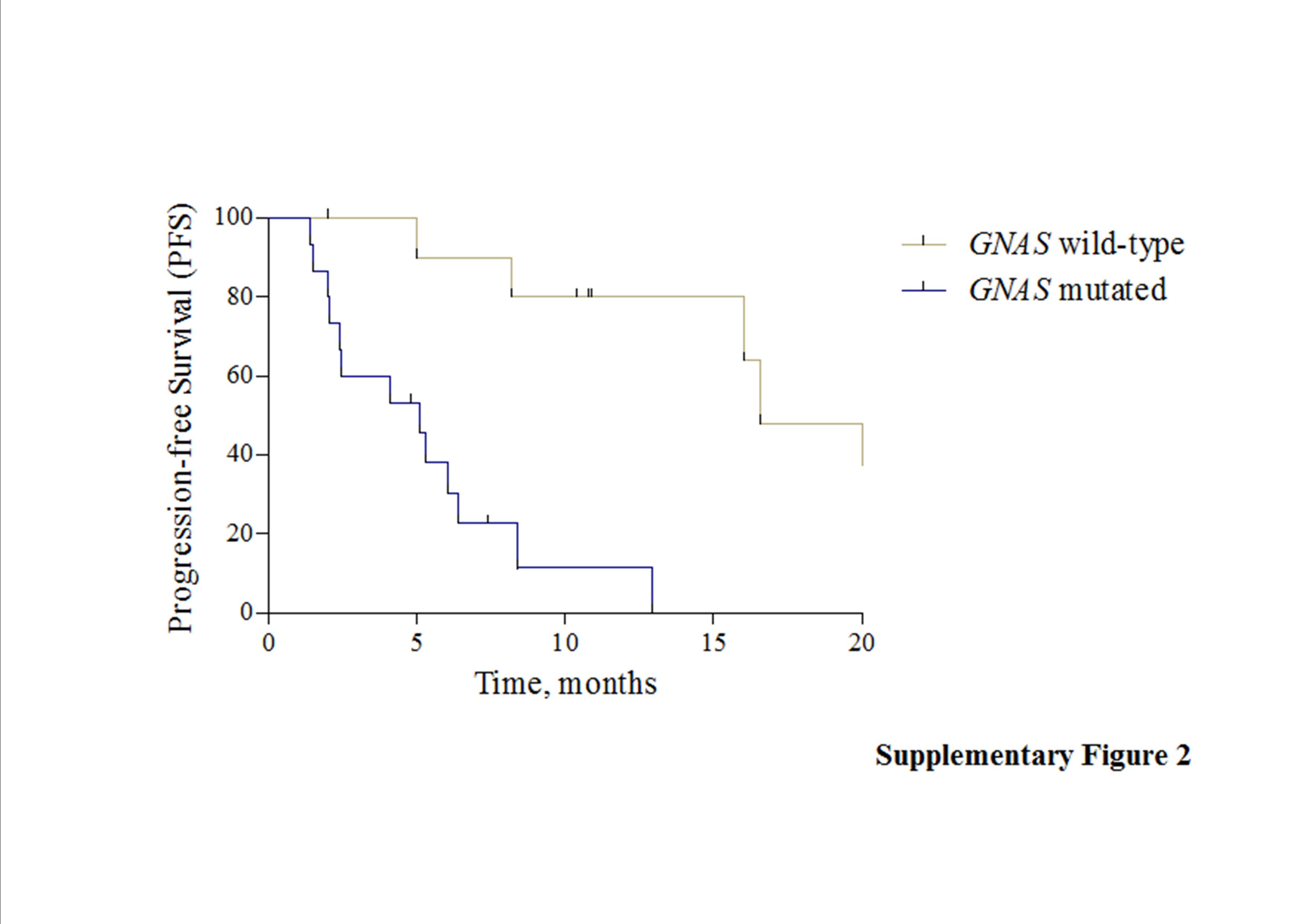

Supplement: Supplementary file 3 — 10.1186/s12967-016-0877-x Comparison of Kaplan-Meier curves for progression-free survival according to GNAS mutational status in both the prospective (metronomic capecitabine and bevacizumab) and the retrospective cohort (FOLFOX-4). [file 12967_2016_877_MOESM3_ESM.jpg]
