# Supplementary material for: GNAS mutations as prognostic biomarker in patients with relapsed peritoneal pseudomyxoma receiving metronomic capecitabine and bevacizumab: a clinical and translational study
Source: J Transl Med. 2016 May 6;14:125. doi: 10.1186/s12967-016-0877-x (PMC4859944; doi:10.1186/s12967-016-0877-x)
Supplement: Supplementary file 4 — 10.1186/s12967-016-0877-x Genomic alterations detected in the 3 GNAS mutated colorectal cancer and matched clinical-pathological data. [file 12967_2016_877_MOESM4_ESM.docx]

**Supplementary Table 1. Genomic alterations detected in the 3 GNAS mutated colorectal cancer and matched clinical-pathological data**

| **ID** | **Mutations detected by NGS** | | | | | | | | | | | | | | |
| --- | --- | --- | --- | --- | --- | --- | --- | --- | --- | --- | --- | --- | --- | --- | --- |
|  | **Tumor content** | ***KRAS* mutation** | **Mutant alleles** | | **Mutant alleles normalized for tumor content** | | **HS** | **GNAS mutation** | | **Mutant alleles** | **Mutant alleles normalized for tumor content** | | **HS** | | **Other mutations (mutant alleles; normalized for tumor content; HS)** |
| 1 | 30% | Q61H | 25% | | 83% | | 166 | R201H | | 20% | 67% | | 134 | | PI3KCA E545K (28%; 93%; 186) |
| 2 | 60% | G12S | 19% | | 32% | | 64 | R201C | | 26% | 43% | | 86 | | PI3KCA E542K (24%; 40%; 80) |
| 3 | 50% | G12C | 37% | | 74% | | 148 | R201H | | 19% | 38% | | 76 | | TP53 E258STOP (25%; 50%; 100) |
| **ID** | **Clinical Data** | | | | | | | | | | | | | | |
|  | **1°LINE** | **OUTCOME** | | **2°LINE** | | **OUTCOME** | | | **Histology** | | | **Site of origin** | | **Sites of metastases** | |
| 1 | Adjuvant FOLFOX | Disease free interval < 6 months | | FOLFIRI plus bevacizumab | | PD; PFS 3 months | | | Mucinous, signet ring variant | | | Right colon | | Isolated peritoneal metastases | |
| 2 | Adjuvant FOLFOX | Disease free interval < 6 months | | FOLFIRI plus bevacizumab | | PD; PFS 3 months | | | Mucinous, signet ring variant | | | Right colon | | Isolated peritoneal metastases | |
| 3 | FOLFOX | PD, PFS 3 months | | FOLFIRI plus bevacizumab | | PD; PFS 1 month | | | Mucinous, signet ring variant | | | Right colon | | Isolated peritoneal metastases | |

| List of abbreviations. HS: heterogeneity score; PD: progression disease; PFS: progression free survival |
| --- |
